# Comments on Structure-based design of antiviral drug candidates targeting the SARS-CoV-2 main protease by Dai et al.(2020)

**DOI:** 10.6026/97320630016432

**Published:** 2020-05-31

**Authors:** Francesco Chiappelli

**Affiliations:** 1 Francesco Chiappelli, Ph.D. Professor Emeritus, UCLA, Center for the Health Sciences, CSUN, Department of the Health Sciences, USA

## Comments:

This article by Dai et al. [1] is an informative piece of writing on the main protease (Mpro, i.e., 3C-like protease, 3-CLpro; E.C.3.4.22.69) of SARS-CoV2, the virus responsible for CoViD-19. Mpro is a cysteine protease enzyme key for the replication and transcription of SARS Corona viral genome. The X-ray structures of the unliganded protease and its complex with an α-ketoamide inhibitor, were the basis for design of α-ketoamide inhibitors for a treatment of SARS-CoV infection a decade ago [2].

Mpro has four enzymatic sites (S1, S1, S2, S4) and a substrate-binding pocket (PDB ID: 2H2Z) that are highly conserved among all Cov's. The present study focuses on Mpro for the development and testing of specific anti-SARS-CoV2 drugs, a clinically relevant question because of the urgent need of antivirals with superior efficiency and safety to contain and control CoViD-19 by suppressing its viral causative agent.

The (+)ss RNA genome of SARS-CoV2 has at least 6 open reading frames, the first of which is about two-third of genome. It translates two polyproteins processed principally by Mpro and into 16 non-structural proteins (nsps). These nsps regulate the production of the four main structural viral proteins (envelope (E), membrane (M), spike (S), and nucleocapsid (N) and for other accessory functional proteins. Therefore, Mpro has a central and critical role in SARS-CoV2 replication.

Two lead compounds (11a and 11b) targeting Mpro with potent anti-SARS-CoV-2 infection activity were designed, synthesized, analyzed and tested in vitro. X-ray crystallography showed that the aldehyde groups of both 11a and 11b covalently bound to Mpro-Cys145. Compound 11a may be superior to 11b with an in vivo pharmacokinetic half-life of following intraperitoneal (5 mg/kg) administration of 4.27 h, a maximal concentration of 2394 ng/mL, a bioavailability of 87.8%, and a satisfactory metabolic stability revealed by a clearance rate of 17.4 ml/min/mg. Biotoxicity studies of compound 11a in vivo in rats and in dogs proffered satisfactory results, indicating that compound 11a may be a good candidate for Phase 1 toxicity trials in normal healthy subjects.

In brief, this is an experimental study, with a small Phase 0 trial component on a laboratory-manufactured compound that blocks the replication of SARS-CoV2, the virus responsible for the CoViD-19 pandemic. The study is important because it is foundational for the development of specific anti-SARS-CoV2 drugs. It has limited immediate value because no attempt was made to establish the biotoxicity, or lack thereof, on human cells in vitro.

## References

[1] Dai W (2020). Science.

[2] Anand K (2003). Science.

